# State-specific Prevalence and Factors Associated With Current Marijuana, ENDS, and Cigarette use Among US Adults With Asthma

**DOI:** 10.1177/1179173X221105783

**Published:** 2022-05-31

**Authors:** Mohammad Ebrahimi Kalan, Zoran Bursac, Rime Jebai, Samane Zare, Wei Li, Prem Gautam, Abir Rahman, Kenneth D Ward, Ziyad Ben Taleb

**Affiliations:** 1 Department of Health Behavior, Gillings School of Global Public Health, 41474 University of North Carolina at Chapel Hill, NC, USA; 2 Lineberger Comprehensive Cancer Center, 41474 University of North Carolina at Chapel Hill, Chapel Hill, Chapel Hill, NC, USA; 3 Department of Biostatistics, Robert Stempel College of Public Health, 5450 Florida International University, Miami, FL, USA; 4 Department of Epidemiology, Robert Stempel College of Public Health, 5450 Florida International University, Miami, FL, USA; 5 School of Medicine, Department of Social Medicine, Population, & Public Health, 8790 University of California Riverside, Riverside, CA, USA; 6 170083 Cabell-Huntington Health Department, WV, USA; 7 School of Public Health, 5415 Memphis University, Tennessee, USA; 8 Department of Kinesiology, College of Nursing and Health Innovation, 12329 University of Texas at Arlington, Arlington, TX, USA

## Abstract

**Background:**

The use of marijuana (MJ), combustible cigarettes (hereafter cigarettes), and electronic nicotine delivery systems (ENDS) is widespread among United States (US) adults and linked to worsening respiratory symptoms, especially among adults with asthma. This study examined state-specific prevalence and factors associated with MJ, ENDS, and cigarette use among US adults with asthma.

**Methods:**

We analyzed data of 41 974 adults aged ≥18 years having self-reported current asthma from the 2018 Behavioral Risk Factor Surveillance System (BRFSS). We reported weighted prevalence to account for complex survey design and performed multivariable logistic regression models to examine factors associated with current use of MJ, ENDS, and cigarettes.

**Results:**

Overall prevalence of current MJ, ENDS, and cigarette use among adults with asthma was 14.5%, 6.6%, and 27.2%, respectively. Our results showed the US states and territories with highest and lowest use prevalence for MJ (California: 23.6% vs Guam: 3.2%), ENDS (Indiana: 12.8% vs North Dakota: 4.0%), and cigarettes (West Virginia: 42.1% vs Guam: 12.3%). Both MJ and ENDS users were more likely to be male, younger, and live in an urban area, but MJ users were more likely and ENDS users less likely to be Non-Hispanic (NH) American Indian/Alaskan Native. Cigarette users were more likely to be older, have at least 1 health condition, and were less likely to be NH Black or Hispanic and college-educated.

**Conclusion:**

Many US adults with asthma use MJ, ENDS, and cigarettes. Our findings provide insights for clinicians about the urgent need for effective interventions to reduce tobacco and MJ use among adults with asthma.

## Introduction

Asthma is a major non-communicable disease, affecting more than 25 million children and adults in the United States (US) with a mortality rate of 10.7 per million.^1^ Substance use, either legal (eg, tobacco products) or illegal (eg, cocaine), can accelerate the decline in lung function and increase life-threatening asthma attacks and asthma mortality.^2,3^ Well-established evidence shows that combustible cigarettes (hereafter cigarettes) smoking can adversely affect clinical, prognostic, and therapeutic outcomes in adults with asthma.^4-6^ Emerging evidence also shows that electronic nicotine delivery system (ENDS) use may worsen asthma symptoms.^7^ Marijuana (MJ) is another apparent popular substance among adults. While the health effects of MJ use on asthma remain controversial among clinicians,^8^ its use can cause an asthma attack leading to hospitalization and even death.^9^

Ongoing legalization of MJ across the US has coincided with the popularity of ENDS use.^10-12^ Therefore, it is crucial to understand the state-specific prevalence of MJ and ENDS use along with cigarette use among adults with asthma to inform clinicians and regulatory bodies. Here, we sought to assess state-specific prevalence and factors associated with MJ, ENDS, and cigarette use among adults with asthma in the US.

## Methods

We analyzed data for participants aged ≥18 years using the 2018 Behavioral Risk Factor Surveillance System (BRFSS). It comprises telephone surveys conducted by all 50 states, the District of Columbia, Puerto Rico, and Guam.^13^ Of the 437 436 respondents, 41 974 reported currently having asthma. We limited our analysis to adults with asthma who provided information on their current use of MJ (n=10 381), ENDS (n=25 280), and cigarettes (n=28 878). Since BRFSS is publicly deidentified state-level data, it is not required to obtain IRB approval.

### Measures

#### Covariates

Assessed covariates included sex, race/ethnicity, education level, age, body mass index, binge and heavy alcohol drinking, residence (urban/rural), chronic obstructive pulmonary disease (COPD), and having ≥1 chronic health condition.^14^ Previous studies^15,16^ found alcohol intake as a trigger for an asthma attack or a risk for developing adult-onset asthma. Therefore, we included the history of alcohol consumption as a potential covariate. Additionally, since COPD and asthma coexist in some patients,^17^ we accounted for having COPD in our analysis (see details for covariates in Table 1 footnote).

**Table 1.** Weighted prevalence and factors associated with current marijuana, ENDS, and cigarette use among adults with current asthma, BRFSS 2018 (n= 41 974)

	Current MJ use (n=10 381)		Current ENDS use (n= 25 280)		Current Cigarette use (n=28 878)	
	(yes vs no)		(yes vs no)		(yes vs no)	
	Weighted%(95%CI)	AOR (95%CI)	Weighted % (95%CI)	AOR (95%CI)	Weighted%(95%CI)	AOR (95%CI)
Overall	14.45 (12.82-16.07)	––	6.5873 (5.90-7.26)	––	27.17 (26.11-28.23)	––
Sex						
Female	51.88(45.63-58.14)^¥^	1 (Reference)	52.84 (47.36- 58.32)^¥^	1 (Reference)	64.30(62.07-66.52)^¥^	1 (Reference)
Male	48.12 (41.86-54.37)	1.82 (1.28-2.60) *	47.16 (41.68- 52.64)	2.38 (1.29-4.38) *	35.70 (33.48-37.93)	1.23 (.86-1.77)
Age, years						
18-24	22.25 (16.68-27.82)^¥^	5.74 (2.77-11.91) *	31.85 (26.79-36.92)^¥^	40.14 (12.95-124.43) *	9.55 (8.16- 10.93)^¥^	1.20 (.50-2.89)
25-34	25.21 (19.98-30.44)	4.67 (2.28-9.56) *	26.32 (21.03-31.61)	9.91 (3.47-28.34) *	21.14 (19.14-23.14)	9.35 (5.43-16.07)*
35-44	18.54 (13.24-23.84)	1.85 (.92-3.72)	16.03 (11.94- 20.12)	9.57 (3.64-25.21) *	19.29 (17.45-21.13)	9.35 (5.66-15.44)*
45-54	13.14 (8.94- 17.35)	1.53 (.75-3.11)	12.76 (9.72- 15.79)	5.44 (2.14-13.83) *	21.19 (19.13-23.25)	6.49 (4.15-10.14)*
55-64	11.63 (8.80-14.46)	1.29 (.66-2.54)	9.79 (7.64- 11.94)	3.80 (1.58-9.14) *	19.79 (18.20-21.38)	4.03 (2.62-6.22)*
≥65	9.22 (5.78- 12.67)	1 (Reference)	3.25 (2.29- 4.21)	1 (Reference)	9.04 (8.01- 10.08)	1 (Reference)
Race/ethnicity						
NH White	48.25 (42.11-54.38)^¥^	1 (Reference)	67.15 (61.63- 72.67)^¥^	1 (Reference)	65.90(63.53-68.27)^¥^	1 (Reference)
NH Black	16.27 (11.17-21.36)	1.79 (1.12-2.88) *	11.04 (7.35-14.72)	.58 (.23-1.44)	15.08 (13.33-16.82)	.44 (.24-.82)*
Hispanic	23.72 (17.66-29.79)	1.60 (.95-2.69)	14.08 (9.11- 19.05)	.30 (.11-.80) *	11.67 (9.73- 13.61)	.44 (.25-.78)*
NH American Indian/Alaskan Native	3.73 (1.73- 5.73)	6.28 (3.02-13.09)*	1.55 (.63- 2.47)	.16 (.03-.83) *	3.20 (2.10- 4.29)	1.34 (.60-3.01)
NH Asian	3.87 (.09-7.64)	.82 (.32-2.09)	1.06 (.23 -1.90)	.89 (.19-4.27)	.57 (.29- .85)	.21 (.04-1.12)
NH Other	4.17 (2.39- 5.94)	1.78 (.87-3.63)	5.12 (3.13- 7.10)	.46 (.17-1.25)	3.58 (3.03-4.14)	.94 (.52-1.69)
BMI						
Normal	31.66(25.88-37.44)^¥^	1 (Reference)	30.26 (25.43- 35.09)^¥^	1 (Reference)	25.81(23.91-27.71)^¥^	1 (Reference)
Underweight	3.49 (1.00- 5.98)	.56 (.19-1.64)	4.82 (2.00- 7.65)	3.80 (1.03-13.99) *	3.37 (2.57- 4.17)	3.00 (1.17-7.72) *
Overweight	31.23 (25.33-37.13)	.95 (.61-1.46)	28.13 (23.40- 32.86)	.73 (.35-1.55)	28.13 (25.98-30.29)	1.12 (.72-1.76)
Obese	33.61 (27.68-39.54)	.56 (.37-.86)*	36.79 (31.17- 42.41)	.74 (.40-1.37)	42.69 (40.30-45.07)	.75 (.51-1.10)
Education						
<high school	18.03(12.21-23.85)^¥^	1 (Reference)	21.27 (15.66- 26.88)^¥^	1 (Reference)	28.97(26.63-31.31)^¥^	1 (Reference)
Highschool	28.47 (23.19-33.76)	.98 (.57-1.69)	35.55 (30.70-40.39)	1.43 (.57-3.57)	33.68 (31.55-35.80)	.67 (.37-1.22)
Some college	37.82 (31.70-43.94)	.96 (.55-1.68)	33.69 (28.69-38.69)	1.85 (.72-4.75)	29.41 (27.40-31.42)	.40 (.22-.72) *
College graduate	15.68 (12.32-19.04)	1.06 (.60-1.85)	9.49 (7.39- 11.59)	.60 (.23-1.57)	7.95 (7.06- 8.83)	.13 (.07-.24) *
Current Cigarette use						
No	48.23 (41.14-55.32)^¥^	1 (Reference)	31.06 (25.10-37.03)^¥^	1 (Reference)	––	––
Yes	51.77 (44.68-58.86)	3.34 (2.21-5.06) *	68.94 (62.97- 74.90)	5.61 (2.77-11.37)*	––	––
Current ENDS use						
No	83.02(78.44-87.60)^¥^	1 (Reference)	––	––	83.31(81.04-85.58)^¥^	1 (Reference)
Yes	16.98 (12.40-21.56)	1.74 (.89-3.39)	––	––	16.69 (14.42-18.96)	4.46 (2.07-9.60)*
Current MJ use						
No	––	––	54.44 (44.41- 64.48)^¥^	1 (Reference)	70.76(66.14-75.38)^¥^	1 (Reference)
Yes	––	––	45.56 (35.52- 55.59)	2.06 (1.09-3.90) *	29.24 (24.62-33.86)	3.27 (2.11-5.08) *
Binge alcohol use ^a^						
No	67.28(61.16-73.40)^¥^	1 (Reference)	69.27 (64.34-74.20)^¥^	1 (Reference)	77.46(75.45-79.47)^¥^	1 (Reference)
Yes	32.72 (26.60-38.84)	3.68 (2.24-6.03) *	30.73 (25.80- 35.66)	1.66 (.74-3.74)	22.54 (20.53-24.55)	2.91 (1.59-5.35) *
Heavy alcohol use ^a^						
No	86.53(81.77-91.30)^¥^	1 (Reference)	88.48 (85.28-91.67)^¥^	1 (Reference)	88.10(86.40-89.81)^¥^	1 (Reference)
Yes	13.47 (8.70- 18.23)	.65 (.31-1.38)	11.52 (8.33-14.72)	1.63 (.56-4.79)	11.90(10.19-13.60)	2.17 (.98-4.81)
COPD ^b^						
No	75.46 (70.72-80.20)	1 (Reference)	71.78 (67.38- 76.19)	1 (Reference)	56.91(54.67-59.16)^¥^	1 (Reference)
Yes	24.54 (19.80-29.28)	1.38 (.90-2.11)	28.22 (23.81-32.62)	1.48 (.71-3.12)	43.09 (40.84-45.33)	4.50 (3.10-6.52) *
≥1 health condition						
No	33.11 (26.86-39.36)	1 (Reference)	29.97 (25.17-34.77)	1 (Reference)	21.27(19.18-23.36)^¥^	1 (Reference)
Yes	66.89 (60.64-73.14)	1.56 (1.05-2.33) *	70.03 (65.23-74.83)	1.54 (.77-3.08)	78.73 (76.64-80.82)	2.40 (1.59-3.61) *
Residence						
Rural	2.75 (1.93- 3.57)^¥^	1 (Reference)	8.11 (3.71- 12.52)	1 (Reference)	8.51 (7.39- 9.63)^¥^	1 (Reference)
Urban	97.25 (96.43-98.07)	1.83 (1.10-3.02) *	91.89 (87.48-96.29)	2.00 (1.01-3.98) *	91.49 (90.37-92.61)	.96 (.67-1.38)

Abbreviations: AOR, Adjusted odds ratio; CI, Confidence interval; MJ, Marijuana; ENDS, Electronic nicotine delivery system; NH, non-Hispanic; COPD, Chronic obstructive pulmonary disease. Sign (¥) denotes a Rao Scot X2 test, which was performed for the univariate analysis of the difference in substance use (marijuana, ENDS, cigarettes) by study covariates. Sign (*) indicates significant AORs.

^a^Binge alcohol drinker (defied as males having 5 or more drinks on 1 occasion, females having 4 or more drinks on 1 occasion), heavy drinkers (defined as males having more than 14 drinks per week and females having more than 7 drinks per week).

^b^The report of having COPD was included in the analysis independently. Those who responded “yes” to “Do you still have asthma?” were considered as currently having asthma.

### Outcome Variables

Adults who reported using MJ at least 1 day in the past 30 days were categorized as current users (yes vs no). Those who responded “*yes*” to questions “*Have you smoked at least 100 cigarettes in your entire life?*” and “*Have you ever used an ENDS…even just 1 time, in your entire life?”* and reported using these products on “*some days*” or “*every day*” at the time of interview were classified as current users of cigarettes and ENDS, respectively.^18^ For cigarettes and ENDS users, those who responded with *“no”* to the aforementioned questions and were not using these products at the time of interview were classified as non-current users of cigarettes and ENDS. Therefore, the outcome for the 2 tobacco products (cigarettes and ENDS) and for MJ were binary coded as “*yes*” (current use) vs “*no*” (non-current use), considering the non-current user group (“*no*”) as the reference group.

### Statistical Analyses

All estimates were weighted using SAS procedures to provide nationally representative and unbiased measures, adjusting for differences in selection probability and nonresponse while accounting for design features. Detailed weighing information for 2018 BRFSS can be found on CDC website.^19^ To examine factors associated with current use (yes vs no [reference group]) of MJ, ENDS, and cigarettes, we applied 3 separate multivariable logistic regression models (1 for each assessed substance) accounting for all above-mentioned covariates. Analyses were conducted in SAS v.9 with a significance level set at α=.05.

## Results

**Figure 1.**
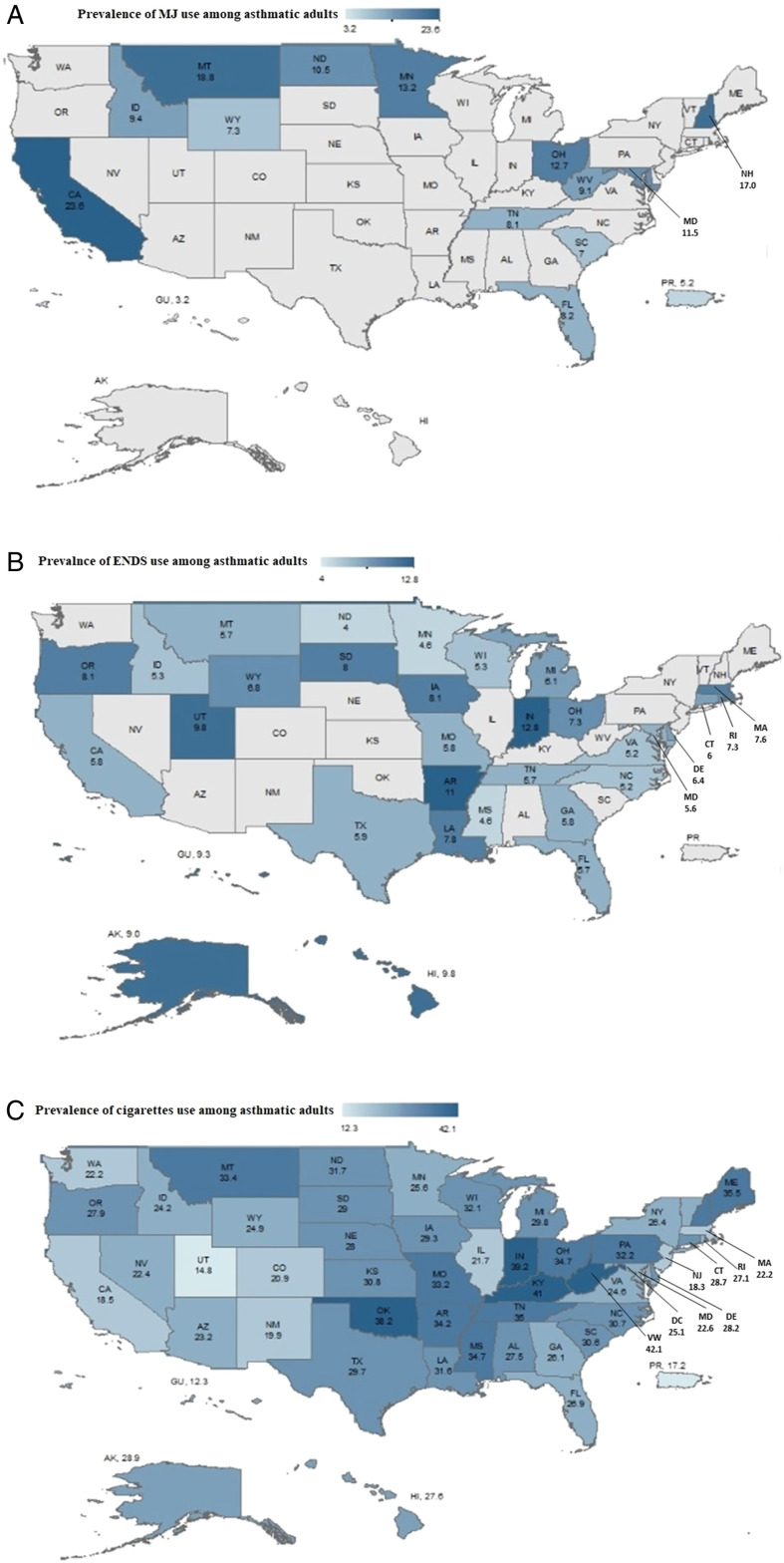
Maps show the percentage of current MJ use (panel A), ENDS use (panel B), and cigarette use (panel C) among adults with current asthma. Note: All states participated in the cigarette module in the 2018 BRFSS survey. Only 31 and 13 states participated in ENDS and MJ use modules, respectively. Data were not available for states indicated in gray.

Weighted prevalence of current MJ, ENDS, and cigarettes use among adults with asthma were 14.5%, 6.6%, and 27.2%, respectively. Figure 1 illustrates a large variation in the use of these products among adults with asthma across the US states and territories, with the highest and lowest use prevalence of MJ (California (CA): 23.6% vs Guam: 3.2%), ENDS (Indiana: 12.8% vs North Dakota: 4.0%), and cigarettes (West Virginia (WV): 42.1% vs Guam: 12.3%).

Adults with asthma who use MJ were more likely to be male, aged 18-34 (compared to ages ≥65), non-Hispanic (NH) Black or NH American Indian/Alaskan Native (compared to NH White), current users of cigarettes and binge drinkers, have at least 1 health condition, reside in an urban area, and less likely to be obese (Table 1). Those who use ENDS were more likely to be male, younger than 65 years (with greatest odds in the 18-24 years old group), current users of cigarettes and MJ, resided in an urban area, and less likely to be Hispanic or NH American Indian/Alaskan Native. Adults with asthma who smoked cigarettes were more likely to be younger than 65 years old, underweight, current users of ENDS, MJ, binge drinkers, reported having a chronic obstructive pulmonary disease (COPD) and at least 1 other health condition, and less likely to be NH Black or Hispanic, and college-educated (Table 1).

## Discussion

This nationally representative study demonstrated that a large proportion of adults with asthma in the US were using MJ, ENDS, and cigarettes with a wide variation across the country. In 2018, 14.5% of adults with asthma were current users of MJ, 6.6% current users of ENDS, and 27.2% current users of cigarettes.^20^ These proportions are higher than what was reported for all US adults in the same year (11% MJ use, 3.2% ENDS use, and 13.7% cigarette use).^18,20^ These differences demonstrate the popularity of these products among adults with asthma and call for urgent actions to curb substance use among adults with asthma.

The variation and differences in characteristics of MJ, ENDS, and cigarettes use patterns among adults with asthma in the US signal the importance of targeted interventions to curb preventable triggers of asthma attacks and improve the quality of life of these patients. For example, findings from our study show that adults with asthma in CA were more likely to use MJ than in other states. This is unsurprising, given that CA has been at the forefront of legalizing MJ use in the US^21^. On the other hand, adults with asthma in WV were more likely to use cigarettes than in other states, which was also expected since smoking is embedded in the culture of a state considered 1 of the top tobacco producers in the US.^22,23^ These geographical differences have important clinical implications since clinicians can provide more product-focused interventions to increase awareness about health effects among adults with asthma based on their state of residence.

Limitations should be noted. The cross-sectional nature of the BRFSS precludes the causal relationships. The BRFSS does not collect MJ data from all US states or territories; therefore, it may not be fully generalizable to the states with different MJ legalization statutes. Although self-reported asthma condition is subject to social desirability bias, the psychometric validity of BRFSS data on health conditions has been confirmed in previous studies.^24,25^ In this study, we focused only on current users of these products (vs. non-users). Unlike other tobacco-specific datasets (eg, PATH), the BRFSS dataset does not include specific variables to confirm former use of tobacco products, thus, we were not able to conduct a more granular analysis of former and experimental use of these tobacco products. Therefore, future studies are warranted to include never users and how it differs from former users among adults with asthma, including adolescents.

A large nationally representative sample of adults with asthma in the US use MJ, ENDS, and cigarettes at higher rates than national estimates. Our findings provide insights for clinicians regarding the exclusive and concurrent use of these products among adults with asthma and associated factors. Additionally, our results will help develop effective public health policies urgently needed in light of the ongoing legalization of MJ and the popularity of ENDS in the US.
